# Dr. Charles Randolph

**Published:** 1934

**Authors:** 


### Obituary

### DR. CHARLES RANDOLPH,
### OF MILVERTON, SOMERSET.

A VERY large circle of patients and friends will mourn the death of Dr. Charles Randolph, which occurred on 14th January, in his eighty-fourth year.

A native of Milverton, son of Dr. Henry W. Randolph, he received his medical education at the Bristol Medical School and Royal Infirmary and at St. Bartholomew's, and after qualifying M.R.C.S.(Eng.) in 1871 and L.R.C.P.(Edin.) and L.M. in 1872 he joined his father and remained in active practice until his retirement in 1931. He always took the keenest interest in his profession. When under forty years of age he was elected President of the West Somerset Branch of the B.M.A., and for forty-five years he was Medical Officer of Health to the Wellington Rural Council, whereas for no less than fifty-five years he acted as Poor Law Officer.

Endowed with an almost superhuman capacity for work, Dr. Randolph was ever willing to expend his last ounce of energy in the interest of his patients, taking practically no recreation, with the exception of an occasional day's hunting; but in spite of the exacting nature of his large and widely-scattered practice he managed to keep himself well informed in all the practical branches of medicine, but he had little opportunity of attending medical meetings or of taking part in discussions, which is a regrettable loss to the profession, since he was an accurate observer and a man of vast experience.

For some years before his death Dr. Randolph suffered much pain from a progressive affection which completely crippled him. He bore his illness with wonderful patience and fortitude.

It is a proud reflection, not only for the Bristol Medical School, but for the whole profession, that one of its members should have left behind him so useful and so unselfish a record as that of Dr. Randolph, who proved himself to be a man of rare personality and one capable of conducting a large general practice for no less a period than sixty years with such ability and untiring devotion to duty that his career may well be regarded as exemplifying the highest traditions of the profession. In addition to his medical skill he had a capacity for taking a genuine personal interest in his patients, which must have accentuated, if possible, his innate desire to be of service. His whole life, in fact, illustrated the force of Browning's remark: "How can man love but what he yearns to help?"

He leaves a widow, two daughters and two sons, and a niece, with whom we deeply sympathize.

